# The Cultural Perception of Abuse in a Multicultural Context and Its Impact on Mental Health: A Case Report

**DOI:** 10.7759/cureus.80779

**Published:** 2025-03-18

**Authors:** Mishalle F Rashid, Richard Leggett, Aleksandra Grzybowska, Bryce Gentry, Ellis Linder

**Affiliations:** 1 Medicine, Edward Via College of Osteopathic Medicine, Blacksburg, USA; 2 Psychiatry, LewisGale Medical Center, Salem, USA

**Keywords:** attention deficit hyperactivity disorder, binge drinking, corporal punishment, cultural dissonance, culturally sensitive psychiatry, mental health, migration stress, personality traits, posttraumatic stress disorder, trauma-informed care

## Abstract

This case report reviews the psychiatric difficulties of a 27-year-old female patient from Botswana with symptoms fitting for posttraumatic stress disorder (PTSD), cluster B personality traits, and attention-deficit/hyperactivity disorder (ADHD). Her case illustrates how corporal punishment, an accepted practice in Botswana, remains in stark contrast to the standards of American society and the psychological consequences that may emerge from such practices. The patient presented with mood instability, anxiety, and recurrent depressive episodes, which were worsened by poor coping mechanisms including binge drinking and emotional dissociation. The patient’s interviews confirmed the presence of PTSD, ADHD, and personality disorder per diagnostic criteria in the DSM-5. She was treated with lamotrigine and Adderall XR, with trauma-informed group therapy and psychoeducation about cultural perceptions of abuse. The patient showed improvement in emotional regulation and investment in the treatment; however, she continued to have difficulties with sleep hygiene and alcohol consumption. This case underlines the importance of culturally sensitive psychiatric care and emphasizes that osteopathic practitioners should further work within the framework of the trauma-informed approach with consideration of cultural influence when managing patients, especially those of migrant backgrounds.

## Introduction

Corporal punishment is a child disciplinary method that is largely deemed controversial and culturally variable [1-2]. While more than 65 countries around the world have banned corporal punishment in all settings, it is still considered a normal part of raising disciplined children in many societies, especially Africa and Central America [1]. For instance, physical punishment is both legally and culturally accepted in Botswana homes and schools [1-3]. Various studies have determined that as many as 92% of children in Botswana receive some kind of corporal punishment in community settings, such as school, and this is considered an acceptable and effective method of discipline [1-2]. In contrast, the United States and a number of other Western countries consider physical punishment abusive due to its long-term potential for psychological harm [4-5]. Research has shown that physical punishment is associated with increased risks for posttraumatic stress disorder (PTSD), anxiety, depression, and personality disorder [6-7].

Migrants who transition from cultures where corporal punishment has been normalized into settings that condemn the practice often experience unique challenges, especially in the realms of physical health, mental health, behavioral development, and academic success [1]. Most of them suffer from cultural dissonance, inner conflict, and an inability to harmonize past experiences with new societal norms [8-9]. Research indicates that many immigrant families, though often raised in cultural contexts where corporal punishment is normalized, express a desire to adopt less injurious disciplinary methods. For example, in one workshop with 70 low-income Mexican and Central American immigrant parents, participants reflected on their own experiences of being hit as children and expressed a desire to avoid similar practices with their children in the US [10]. Yet, some continue corporal punishment because it is a part of their culture. The children internalized these measures as psychological results and could not often distinguish between discipline and abuse [11-12].

The psychological outcomes of corporal punishment have been widely examined. For example, one 2016 meta-analysis of 75 studies involving more than 160,000 children demonstrated that spanking was associated with increased externalizing behaviors and reduced mental health outcomes [4]. However, there are still gaps in understanding how such findings apply to individuals from non-Western backgrounds where corporal punishment may carry different cultural connotations [12-13]. Gaps continue to exist regarding the ways in which migrants from corporal punishment-accepting societies make their transition to nations with more stringent anti-abuse norms.

This case report describes the unique mental health presentation of a 27-year-old female patient from Botswana who was diagnosed with PTSD, attention-deficit/hyperactivity disorder (ADHD), personality psychopathology, and maladaptive coping mechanisms. Her case underlines the complex interplay of cultural norms, migration, and mental health. This report aims to characterize the need for culturally sensitive psychiatric care in helping patients process and reconcile their experiences within a new cultural framework. This report adds to the limited literature on the way cultural perceptions of corporal punishment impact mental health outcomes for migrant populations.

## Case presentation

Patient demographics

The patient is an unemployed 27-year-old white female born in Botswana who immigrated to the United States at age 10. The patient was referred to and admitted to an Intensive Outpatient Program (IOP) for the management of mood instability, anxiety, and depression. Written informed consent to publish this case was obtained and provided electronically by the patient after reviewing it with the lead physician, Dr. Richard P. Leggett.

History of present illness

The patient presented to our IOP with a history of mood swings, anxiety, and recurring depressive episodes with exacerbation at night. She also gave a history of nihilistic thinking as well as engaging in self-batter as an adolescent during which she would cut herself but was never hospitalized. She reported an incredibly abusive childhood in Botswana, characterized by frequent verbal and physical punishment such as a wooden spoon hitting her body or pinching her in public. These disciplinary measures were normalized within her community but later conflicted with U.S. cultural norms and thus created internal conflict and delayed recognition of the emotional harm. When she was in high school, her parents divorced, and she experienced significant verbal and emotional abuse from her mother. Her history included two suicide attempts without hospitalization or psychiatric evaluation during adolescence and a binge drinking pattern that emerged in adulthood as a coping mechanism for managing emotional distress. She also continued to endorse chronic difficulties in emotion regulation, relationships, and impulsivity consistent with her diagnosis.

Diagnosis and management

The patient underwent psychiatric assessment during her time in the IOP program, including serial evaluations of her mental status. The patient was noted to have hyperverbal speech, tangential thought processes, and mood lability. Based on clinical presentation, history, and DSM-5 definition, the patient was diagnosed with multiple comorbid conditions. The patient was diagnosed with PTSD due to symptoms of recurrent nightmares, hypervigilance, avoidance of emotional triggers, cluster B personality traits due to borderline and dependent personality organizations, ADHD due to observed impulsivity and emotional dysregulation, and Binge Drinking Disorder due to patient admitted patterns of excessive alcohol use associated with stress. Formal diagnostic scales such as the PTSD Checklist for DSM-5 (PCL-5) were not administered but are recommended for future assessments in quantifying symptom severity.

The patient was prescribed lamotrigine 200 mg daily as indicated for mood stabilization and taken cautiously to avoid the risk of Stevens-Johnson Syndrome during its titration. For symptomatic management of ADHD, she was started on dextroamphetamine/amphetamine XR 30 mg daily, although consideration of avoiding doses after 2 P.M. was recommended to enhance sleep hygiene. For breakthrough symptoms of ADHD, she was prescribed dextroamphetamine/amphetamine IR 15 mg PRN. The patient was recommended group therapy for coping skills, mindfulness training, and dealing with dissociation and masking behavior. Additionally, she began psychoeducation to support the patient in working through cultural differences in discipline versus abuse. Video games and writing were recommended as hobbies to engage in, as the patient reported previous enjoyment in these activities. Recommendations were provided to the patient on sleep hygiene, such as regular bedtime and restriction of caffeine. Additionally, the patient was advised to lessen the consumption of alcohol in order to decrease its effect on mood and sleep. The patient was educated on the necessity of treatment plan adherence and the risks of acute cessation of medications outside of a clinical setting. She clearly understood and agreed to the plan of treatment, volunteering her informed consent.

Follow-up and outcomes

The patient was scheduled for weekly follow-ups during the six- to eight-week IOP program and referred to outpatient care upon discharge. The patient displayed improvement in mood stabilization, though residual anxiety and depressive symptoms remained. There was improvement in engagement in hobbies and participation in group therapy, though sleep regulation and alcohol intake continued. The patient was further followed up with posttreatment diagnostic tools such as repeat mental status examinations, with formal outcome measures (PHQ-9 scores). Additional formal outcome measures, for example, the use of the PCL-5 or GAD-7, are recommended for future follow-ups.

Recovery instructions

The patient was advised to continue medications as prescribed: lamotrigine 200 mg daily, dextroamphetamine/amphetamine XR 30 mg daily, and dextroamphetamine/amphetamine IR 15 mg PRN. The patient was also recommended to continue participation in group therapy sessions. Additionally, the patient was advised to allow her father to assist her in managing her finances. Finally, the patient was encouraged to maintain a dream book to assist in analyzing reoccurring nightmares.

Outcomes

**Figure 1:**
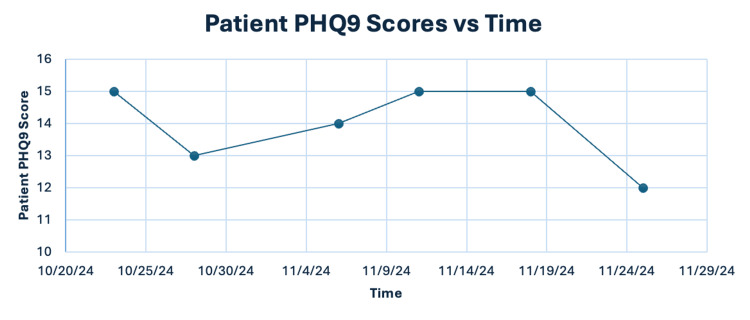
Line graph depicting patient PHQ9 scores over time.

This patient demonstrated significant improvement during her time in the IOP. Figure 1 shows this improvement based on her PHQ-9 scores throughout this program. We first see the positive effect of treatment at the beginning of her case, between October 23, 2024, and October 28, 2024. Afterward, from November 6, 2024, to November 18, 2024, improved awareness of trauma resulted in an acutely worsening trajectory. Improvement was noted from November 18 to November 25, 2024, where the patient demonstrated a 20% reduction in PHQ-9 score, consistent with her observed benefit. The patient noted subjective improvement in mood and self-awareness. Binge drinking episodes reduced in frequency but had not completely stopped. Sleep hygiene remained poor, although the patient demonstrated a desire to institute changes as advised. The patient was more aware of her upbringing amidst cultural differences between Botswana and the United States and the influence rectifying the two had on her mental health; therapy provided a framework in which she worked through experiences.

## Discussion

The presented case demonstrates a very complex interaction between cultural norms, migration, and mental health, therefore providing an opportunity for valuable insights into challenges associated with a multicultural background. The patient's presentation is a good example of how culturally perceived corporal punishment and change in society after migration can affect mental health. Her unique cultural context underlines the need for cultural sensitivity in psychiatric and osteopathic management.

Context of patient management

The patient's upbringing in Botswana normalized physical punishment as an acceptable disciplinary tool. National surveys have reported that 92% of children in Botswana may be subjected to corporal punishment at school, while cultural acceptance indicates a greater prevalence in the home setting [1-2]. Such practices are entrenched in both cultural and legal systems; for example, corporal punishment is allowed under the Children's Act of Botswana, reflecting broader societal acceptance [3]. However, these disciplinary actions have become increasingly acknowledged as being truly harmful psychologically. Meta-analyses of more than 160,000 children reported that corporal punishment is associated with a 54% increased risk of externalizing behaviors as well as increased risks for PTSD, depression, and anxiety [4-5]. Migration exacerbated the psychological effects of the patient's childhood experiences. Acculturation stress, or the psychological strain of adjusting to a new culture, has been associated with poorer mental health in migrant groups. Numerous studies of migrants in the United States have indicated that those from collectivist cultures, such as Botswana, were more vulnerable to depression and anxiety brought on by perceived conflicts between cultural values and societal norms in their new environment [6-7]. In this patient's situation, moving into the United States' paradigm, in which corporal punishment is largely considered abusive, created an internal conflict that postponed her recognition of trauma [11].

Significance of presentation and outcomes

The patient's case illustrates several important considerations. While corporal punishment is legally and culturally supported in Botswana, its outcomes are not harmless. Longitudinal studies have shown that early exposure to physical punishment increases the risk for PTSD and other mental health disorders, even in cases when this form of treatment had been perceived as being culturally appropriate [8-9]. The effect on the patient could be seen in recurrent nightmares, emotional dysregulation, and difficulties in interpersonal relationships. The patient's case serves to illustrate the role of cultural dissonance. Immigrants often undergo psychological tension when societal expectations run in contrast to deeply held cultural values. More so, it reiterates the notion that immigrants to the United States often have difficulty reconciling traditional disciplinary practices with new societal expectations. This broader trend is underlined by the resistance of the patient to define experiences from her childhood as abusive and the need for psychoeducation to bridge cultural gaps. This case offers important insights into the interplay between culture and trauma in mental health, including delayed recognition of trauma (cultural attitude of acceptance towards corporal punishment delayed the patient's realization of the emotional trauma she had suffered), maladaptive coping mechanisms (binge drinking and dissociation as coping strategies are well documented in the literature among trauma survivors but, in her case, are compounded by cultural stigmas surrounding mental health in Botswana [12-13]), and integration of dual cultural identities (highly conflictual cultural norms in navigating her self-concept and progress in therapy). The identified characteristics further highlight the necessity of psychiatric treatment being individualized and molded around the patients' cultural backgrounds. The incorporation of psychoeducation and culturally sensitive therapies can help patients manage conflict and facilitate more adaptive ways of coping.

Clinical implications and applicability

This case has several implications for physicians and mental health practitioners. The holistic approach central to osteopathic medicine - a philosophy that places great importance on the integration of mind, body, and environment - is particularly well-suited for patients navigating dual cultural identities. For example, culturally adaptive psychoeducation (educating the patient on the psychological effects of corporal punishment while validating her cultural identity) was a critical aspect of her care. Such interventions align with osteopathic principles including respecting the patient’s lived experiences [13] in combination with trauma-informed care (recognizing the patient’s delayed understanding of her trauma required a nonjudgmental, trauma-informed approach that acknowledged her cultural background [14]). Other interventions included focusing on functional outcomes including recommendations to improve sleep hygiene, reduce alcohol consumption, and engage in supportive relationships targeted functional improvements in alignment with osteopathic principles [15]. This patient’s experience also underscores the need for training mental health professionals to incorporate cultural competence into diagnostic and therapeutic frameworks. Future clinical guidelines should emphasize the integration of cultural, psychological, and somatic factors to provide more effective care [16].

This case contributes to an emerging body of literature related to the psychological consequences of corporal punishment and the distinct struggles of migrant populations. Although previous research has identified the lasting impact of physical punishment [4-5], a knowledge gap remains regarding the analysis of how such punishment affects migrants in transitioning cultures. This report helps to fill that knowledge gap by demonstrating the ways in which cultural practices shape the identification of trauma and subsequent mental health treatment. This paper also underlines the wider ramifications of the process of acculturative stress on migrant mental health. Immigrants who migrate from corporal punishment-accepting countries often experience higher vulnerabilities to anxiety and depression in balancing these conflicting norms [6-7]. The patient's history also illustrates how a combination of psycho-education with culturally sensitive therapies can effectively help overcome these challenges.

## Conclusions

This case demonstrated how culturally sensitive psychiatric care is important in the management of patients' mental health needs while negotiating conflicting cultural values. Clinicians can address psychological effects attributed to corporal punishment within the patient's culture through the use of trauma-informed, holistic care approaches that help patients reconcile their experiences and improve their outcomes. The insights from this case contribute to a better understanding of the interaction between culture, trauma, and mental health and may provide a conceptual framework for liaison psychiatry.
